# Firefighters’ absorption of PAHs and VOCs during controlled residential fires by job assignment and fire attack tactic

**DOI:** 10.1038/s41370-019-0145-2

**Published:** 2019-06-07

**Authors:** Kenneth W. Fent, Christine Toennis, Deborah Sammons, Shirley Robertson, Stephen Bertke, Antonia M. Calafat, Joachim D. Pleil, M. Ariel Geer Wallace, Steve Kerber, Denise Smith, Gavin P. Horn

**Affiliations:** 1 Division of Surveillance, Hazard Evaluations, and Field Studies, National Institute for Occupational Safety and Health (NIOSH), Centers for Disease Control and Prevention (CDC), Cincinnati, OH USA; 2 Division of Applied Research and Technology, NIOSH, CDC, Cincinnati, OH USA; 3 Division of Laboratory Services, National Center for Environmental Health, CDC, Atlanta, GA USA; 4 National Exposure Research Laboratory, Office of Research and Development, U.S. Environmental Protection Agency, Research Triangle Park, NC USA; 5 Firefighter Safety Research Institute, Underwriters Laboratories, Columbia, MD USA; 6 Skidmore College, Saratoga Springs, New York, NY USA; 7 Illinois Fire Service Institute, University of Illinois at Urbana-, Champaign, IL USA

**Keywords:** Breath, Urine, Biomarker, Firefighter, PAH, Benzene

## Abstract

To better understand the absorption of combustion byproducts during firefighting, we performed biological monitoring (breath and urine) on firefighters who responded to controlled residential fires and examined the results by job assignment and fire attack tactic. Urine was analyzed for metabolites of polycyclic aromatic hydrocarbons (PAHs) and breath was analyzed for volatile organic compounds (VOCs) including benzene. Median concentrations of PAH metabolites in urine increased from pre-firefighting to 3-h post firefighting for all job assignments. This change was greatest for firefighters assigned to attack and search with 2.3, 5.6, 3.9, and 1.4-fold median increases in pyrene, phenanthrene, naphthalene, and fluorene metabolites. Median exhaled breath concentrations of benzene increased 2-fold for attack and search firefighters (*p* < 0.01) and 1.4-fold for outside vent firefighters (*p* = 0.02). Compared to interior attack, transitional attack resulted in 50% less uptake of pyrene (*p* = 0.09), 36% less uptake phenanthrene (*p* = 0.052), and 20% less uptake of fluorene (*p* < 0.01). Dermal absorption likely contributed to firefighters’ exposures in this study. Firefighters’ exposures will vary by job assignment and can be reduced by employing a transitional fire attack when feasible.

## Introduction

Structure fires typically involve furnishings and other items made of both natural and synthetic materials. These fires can produce hundreds of combustion byproducts, including benzene, polycyclic aromatic hydrocarbons (PAHs), acid gases, hydrogen cyanide, aldehydes, inorganic gases, and halogenated compounds [1–5]. Several of these compounds (e.g., benzene, benzo[a]pyrene, formaldehyde) are known or suspected human carcinogens [6–8]. Epidemiology studies suggest that firefighters have increased risk for numerous types of cancer [9–13] and the International Agency for Research on Cancer (IARC) classified occupational exposure as a firefighter to be possibly carcinogenic to humans (Group 2B) [14]. Firefighters’ exposure to chemical carcinogens, particularly those associated with byproducts of combustion, has been postulated as a contributor to this increased risk [9].

Firefighters usually use self-contained breathing apparatus (SCBA) when conducting interior operations like fire attack (i.e., suppressing the seat of the fire) or search and rescue. However, firefighters may not always wear SCBA during exterior operations, such as incident command (i.e., directing and supervising the response), pump operation, or outside ventilation (i.e., opening walls or roof in an attempt to clear smoke from the structure). In addition, firefighters may remove SCBA during overhaul, which is the period of the response after fire suppression when firefighters search for smoldering items inside the structure. Although the inhalation route is protected by use of SCBA, the potential for dermal exposure still exists. Studies have found PAH particulates under firefighters’ protective ensembles (i.e., turnout gear) and contaminating the skin following fire responses [15–20] and PAHs can be readily absorbed through skin [21–24].

While many studies on firefighters have focused on PAHs and other solid-phase contaminants, few studies have examined the penetration of vapors into the interior space of the turnout gear. Wingfors et al. [15] found that naphthalene, the most volatile PAH, more readily penetrated the protective barriers of turnout gear than less volatile PAHs. Other volatile chemicals like benzene may also penetrate turnout gear. One component of turnout gear that likely provides very little attenuation for vapors is the hood, which is typically made of a couple layers of porous fabric, such as Nomex® (DuPont, Wilmington, DE).

Exposure of the neck to chemicals during firefighting could contribute to total body burden. Chemicals that deposit onto skin generally penetrate thin skin (e.g., neck) faster than thick skin (e.g., plantar foot arch) [23, 25, 26]. While non- or semi-volatile compounds can readily deposit onto skin, volatile organic compounds (VOCs) typically remain in vapor phase. However, small amounts of VOCs can partition to solid phase and condense to the skin where they may be available for biological uptake. For example, up to 1% of benzene vapor may be absorbed directly through skin [27–29]. Therefore, firefighters could absorb VOCs into their bodies even when wearing SCBA and turnout gear.

Several studies have documented increased excretion of PAH and/or benzene metabolites in urine or breath following structural firefighting [15, 16, 18, 30–32]. In many of these studies, both dermal and inhalation routes could have contributed to the systemic levels. However, in our previous study that involved a room and contents fire [18], firefighters wore their SCBA throughout the exercise. Firefighters in this study showed statistically significant increases in exhaled breath concentrations of benzene, styrene, and naphthalene and increased excretion of PAH metabolites following firefighting [18, 33]. The PAH metabolites were measured using an enzyme-linked immunosorbent assay (ELISA), which is not specific, but correlates well (r = 0.89) with the sum of metabolites of four PAHs (naphthalene, fluorene, phenanthrene, and pyrene) measured by gas chromatography-mass spectrometry in exposed workers’ urine [34]. Other studies of firefighters have documented post-fire increases in metabolites of phenanthrene, fluorene, and pyrene [15, 16].

These past studies indicate that firefighters’ will absorb toxic compounds into their bodies during live fire responses. While studies exploring biological levels of these compounds by job assignment are lacking, our group recently reported that firefighters assigned to fire attack and search and rescue operations had significantly higher personal air concentrations of total PAHs and benzene and higher levels of PAHs on their hands than firefighters assigned to other jobs (e.g., overhaul, command, outside ventilation) [5, 20]. Exposures could also vary by attack tactic. Two types of tactics that may be used for suppression of structural fires are: (1) interior attack and (2) transitional attack. During interior attack, firefighters enter the structure and immediately search for and apply water to the fire. In transitional attack, firefighters attempt to get water on the fire as soon as possible by identifying a window or other opening near the fire and applying water from outside the structure before transitioning to interior attack. To our knowledge, no studies have examined the exposures experienced by firefighters employing different tactics.

The purpose of this study was to investigate the biological uptake of PAHs and VOCs (i.e., benzene, toluene, ethylbenzene, xylenes) using urine and breath samples, respectively, following controlled residential fire responses and to compare the levels by job assignment and fire attack tactic. Urinary concentrations of PAH metabolites were quantified using both ELISA and mass spectrometry, and the results compared. The findings in this paper may be useful to the fire service in understanding firefighters’ systemic exposures to combustion byproducts and identifying ways to reduce those exposures.

## Materials and methods

### Participants

This study was reviewed for compliance with all applicable policies for ethical conduct and the protection of human subjects and received approval by the Institutional Review Boards at the University of Illinois and the National Institute for Occupational Safety and Health (NIOSH). Participants were recruited through a nationwide multimedia effort along with a focused effort by a statewide network of firefighters who teach and train at the University of Illinois Fire Service Institute. Participants provided informed written consent indicating that they understood and voluntarily accepted the risks of participation. Firefighters with any known cardiovascular disease, or who used tobacco, were younger than 18 or older than 55 years of age, or pregnant were excluded from the study. Participants were also requested to avoid eating char-grilled or smoked foods 24-h before and during each study day. Power calculations (80% power at 0.1 significance level) were conducted using variance estimates from Fent et al. [18] to estimate the sample size (*n* = 31 per group) to detect a 25% difference in PAH metabolite concentrations in urine. In total 41 (*n* *=* 41) firefighters (37 male, 4 female) participated in this study; five dropped out and were subsequently replaced.

### Study design

The study design is described in detail elsewhere [5, 20, 35] and summarized via a flow chart in supplemental materials (Fig. S1). Briefly, using a repeated-measures design, we grouped study participants into crews of 12 firefighters and deployed each crew to a pair of fire scenarios using 2 different fire attack tactics. Six fire scenarios were suppressed using an interior fire attack and 6 fires were suppressed using a transitional fire attack. Half of the crews responded to the first fire using interior attack and the second using transitional attack, while the other half of the crews initially used transitional attack followed by a second scenario with interior attack. The 12 firefighters were assigned in pairs to 6 fireground jobs, encompassing inside operations (fire attack, and search and rescue), outside operations (command, pump operator, and outside ventilation), and overhaul operations (two pairs of firefighters). After completing the first two fire exercises (on two consecutive days), the firefighters had a break of several days, were reassigned to new positions, and then completed the next two fire exercises on consecutive days. Firefighters wore National Fire Protection Association 1971-compliant protective ensembles, including a double-layer Nomex® hood [36]. The turnout jackets had standard zipper and/or hook and loop interface enclosures.

The fire attack team pulled a hose line from the engine and extinguished all active fire. The search team forced entry to the structure, then searched for and rescued two simulated occupants (75 kg manikins). The outside vent team deployed ladders to the structure and used hand tools to create openings at the windows and roof. Command/pump teams established incident command and operated the pump panel. During fire suppression, two teams of overhaul firefighters either pulled a backup line (backup/overhaul) or set up as a rapid intervention team (RIT/overhaul) on the fireground. After fire suppression was complete, the overhaul firefighters entered the structure to search for smoldering items and remove drywall and furniture from the structure.

The fire scenarios took place inside a 111 m^2^ wood frame residential structure [20]. Investigators ignited fires in two of the bedrooms, which were appointed with modern furnishings. After ignition, the research team allowed the fires to grow until the rooms approached flash over (typically 4–5 min) and then dispatched the firefighters. After each fire, the drywall and furniture were replaced with identical items (from a single source).

Firefighters performing inside and overhaul operations were required to breathe from their SCBA upon entering the structure. For outside operations, firefighters were instructed to use their SCBA as they normally would. Generally, the incident commanders and pump operators did not breathe from SCBA during the exercises. Some outside vent firefighters did breathe from SCBA during operations; however, SCBA usage was not documented for this group (i.e., videography failed to capture SCBA use for these firefighters). Upon completion of firefighting duties, participants doffed their gear and reported to a data collection room where their neck and hand skin were sampled and cleaned [20]. Afterwards, they rehabbed and recovered for ~30 min and then showered.

### Urine sampling and analysis

Urine samples were collected from firefighters pre-firefighting and 3-h post-firefighting for all scenarios (*n* = 142 person-events; one firefighter dropped out of two scenarios) as previous work indicated that 3-h may represent peak excretion of PAH biomarkers [18]. For half of the interior attack scenarios, urine samples were collected 6-h, 12-h, and 23-h post-firefighting (*n* = 36 person-events) to further explore excretion profiles. Participants were given sterile 120-ml specimen collection cups. We aliquoted all urine samples into labeled tubes and stored them on dry ice while in the field. Samples were then stored at −20 °C for those pending PAH metabolite measurements and at −80 °C for those pending cotinine and creatinine measurements. The urinary PAH-metabolite assay was performed using a modified version of a commercial ELISA (PAH RaPID Assay®, Strategic Diagnostics Inc., Newark, DE) to detect PAH metabolites in aqueous samples [34]. The concentrations are reported as phenanthrene kit equivalents with a method detection limit of 20 ng/mL.

Banked urine samples (*n* = 96 person-events) from the attack, search, outside vent, and backup/overhaul firefighters were shipped to the CDC National Center for Environmental Health to be analyzed for hydroxylated PAH (OH-PAH) metabolites. Briefly, conjugated OH-PAH metabolites in urine (100 µL) were enzymatically deconjugated and analytical determination of the target OH-PAHs was performed by online solid phase extraction coupled with high performance liquid chromatography-isotope dilution tandem mass spectrometry (HPLC-MS/MS). Limits of detection (LODs) ranged from 8 to 90 ng/L, depending on the analyte [37].

Creatinine was measured using a Vitros Autoanalyzer (Johnson & Johnson, New Brunswick, NJ) with a Vitros CREA slide. Cotinine, a metabolite of nicotine, was measured using Diagnostic Products Corporation (Siemens Corporation,Washington, DC) Immulite® 2000 analytical platform. Cotinine concentrations were used to confirm current non-tobacco use status of the participants and to quantify possible exposure to environmental tobacco smoke (ETS), which can be a source of PAH exposure [38]. The vast majority of urine samples (87%) had cotinine levels consistent with non-tobacco use status and no ETS exposure (<10 ng/mL).

### Exhaled breath sampling and analysis

Exhaled breath samples were collected from firefighters before, immediately after, and 1 h after each fire (*n* = 142 person events) as previous work suggested that peak levels right after firefighting returned to baseline within an hour [18]. Collections took place inside an empty bay at the fire training facility. Firefighters were instructed to take a deep breath in and then forcefully exhale their entire breath into the Bio-VOC™ sampler (Markes International, Inc., Cincinnati, OH), which serves to collect the final 129-mL of breath. The collected air was pushed through Markes thermal desorption tubes (Carbograph 2TD/1TD dual bed tubes). Firefighters assigned to attack and search were instructed to keep their SCBA masks on until they reached the sample collection area to eliminate the potential for inhalation exposure to VOCs off-gassing from contaminated clothing and equipment. These particular firefighters took a deep breath in while still on air, removed their regulator, and then exhaled into the sampler. Other firefighters removed their SCBA in the doffing area before coming to the breath collection area. The thermal desorption tubes were capped and stored at −20 °C until shipment to the U.S. Environmental Protection Agency analytical laboratory.

The method used to analyze the breath samples is described in detail elsewhere [39]. Method detection limits (MDLs) ranged from 0.11 ng/tube for ethylbenzene to 0.70 ng/tube for benzene. The ng on tube was converted to ng/L by dividing by the total breath volume collected (129 mL) and results are reported as parts per billion volume (ppbv).

### Data analysis

Statistical comparisons were performed for urinary PAH metabolites and breath concentrations of VOCs measured among attack and search firefighters by attack tactic. We compared post-exposure biological levels (at different time points) to pre-exposure levels for individuals in each job assignment to determine the most exposed jobs. We also compared the change in breath concentrations of benzene among attack and search firefighters with unprotected airway exposure to a smoke plume before donning SCBA to those without that exposure. Lastly, spearman correlation analysis was performed between the HPLC-MS/MS and ELISA PAH metabolite results at different time points.

We used values calculated using instrumental readings for the 0.77% of OH-PAH metabolite results (HPLC-MS-MS) and 35% of the non-specific PAH metabolite results (ELISA) that were <LODs. Although these values did not meet our standard of detection, they likely provided more reliable estimates for censored data than using imputation approaches. Ethylbenzene, total xylenes, and styrene were non-detectable in 3.7, 4.2, and 8.4% of the breath samples. In some cases, the values calculated using instrumental readings for these analytes were negative. Thus, we estimated breath concentrations <LOD using ordered imputations and Q–Q plots as described in Pleil et al. [40, 41]. This method relies on plotting the natural log of the compound concentrations (minus non-detects) versus the Z-scores to obtain a linear best fit equation. This equation is then used to impute values for the samples with concentrations <LOD by plugging the corresponding calculated Z-scores into the obtained equation.

All analyses were performed in R version 3.4.3. Quartiles were used to summarize data. Box-plots were created with lower quartile, median and upper quartiles indicated with the box and whiskers extending to the min and max of the distribution. Mixed linear models, which took into account the repeated measures by firefighter (time [pre, post] and tactic [interior, transitional]), were performed on the log of the exposure measurements. Histograms of the residuals were examined and the normality assumption was considered to be valid.

## Results

### Urinary excretion of PAHs after firefighting

**Fig. 1:**
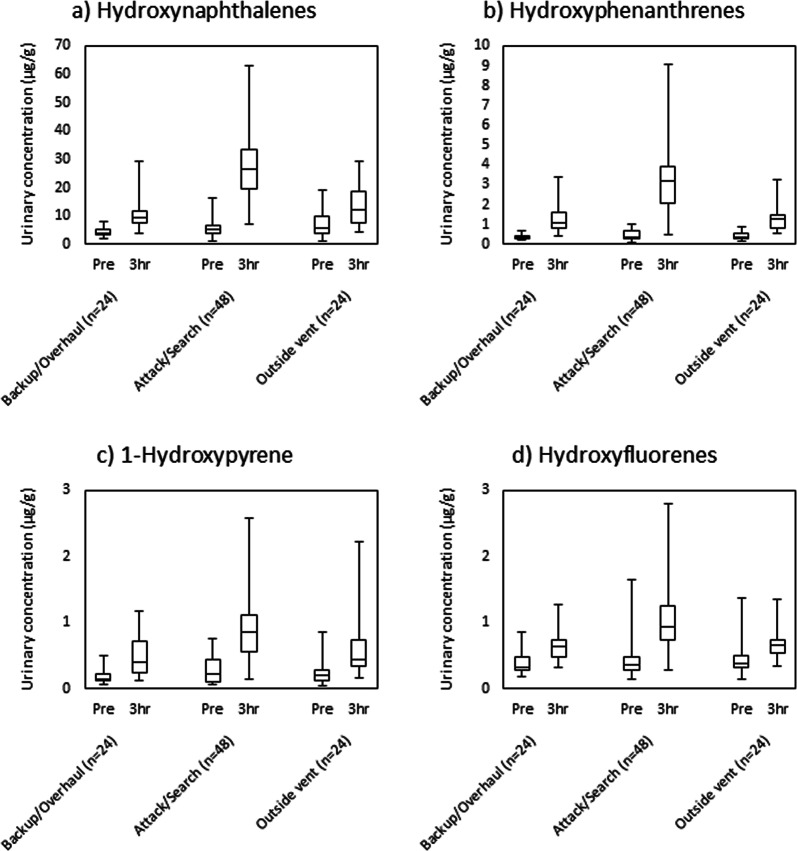
Urinary concentrations (µg/g) of **a** hydroxynaphthalenes, **b** hydroxyphenanthrenes, **c** 1-hydroxypyrene, and **d** hydroxyfluorenes by collection time point for 24 firefighters (distributed evenly among 3 crews, 4 fires per crew) assigned to attack, search, backup/overhaul, or outside vent. Note that firefighters were reassigned after first two fires. All 3-h post-firefighting levels were significantly higher than pre-firefighting levels (*p* < 0.01)

Figure 1 provides the results of the specific OH-PAH metabolites over time by job assignment normalized by creatinine. Note that these analyses were done on a subset of the urine samples (collected from 96 person-events). Metabolites for each parent compound were summed together to simplify the analysis. Attack and search firefighters were grouped together because their urinary PAH metabolite concentrations over time were similar (e.g., comparison of pre to 3-h post change in OH-PAH metabolites, *p* > 0.25). In all cases, median concentrations of the OH-PAH metabolites increased significantly (*p* *<* 0.01) from pre-exposure to 3-h post exposure. The magnitude of this change was greatest in firefighters assigned to attack and search (ranging from 2.4-fold increase for hydroxyfluorenes to 6.6-fold increase for hydroxyphenanthrenes). Of the different metabolites, hydroxynaphthalenes had the largest pre to 3-h unit increase (median increase of 27.2 µg/g creatinine in the attack and search firefighters). It is important to note that the firefighters’ pre-firefighting median concentrations of OH-PAH metabolites were 1.1 to 2-fold higher than the general non-smoking 20–49 year-old population medians (as measured by the National Health and Nutritional Examination Survey [NHANES]) for all but 2-hydroxynaphthalene [42]. Thus, many firefighter participants began each study day with slightly elevated OH-PAH metabolite concentrations.

On the days when urine samples were collected up to 23-h post firefighting, 3-h appeared to represent the peak excretion with the exception of 1-hydroxypyrene for the attack and search firefighters whereby peak excretion occurred at 6-h (Table 1). The attack and search firefighters’ urinary concentrations of hydroxynaphthalenes, hydroxyphenanthrenes, and 1-hydroxypyrene remained significantly elevated 23-h after firefighting (*p* < 0.01).

**Table 1:** Median urinary concentrations of OH-PAH metabolites (µg/g) in 24 firefighters (distributed evenly among 3 crews) assigned to attack, search, outside vent, or backup/overhaul who were sampled for 23 h after a single interior fire attack scenario

Metabolite^a^	Collection period	Attack / search (*n* = 12)^b^	Outside vent (*n* = 6)^b^	Backup / overhaul (*n* = 6)^b^
Hydroxynaphthalenes	Pre	4.8	5.4	3.7
	3-h	32*	14*	9.3*
	6-h	16*	8.8*	6.2*
	12-h	8.0*	6.4	5.0*
	23-h	6.4*	5.4	4.2
Hydroxyphenanthrenes	Pre	0.26	0.28	0.36
	3-h	3.1*	1.3*	1.1*
	6-h	2.2*	0.83*	0.73*
	12-h	1.0*	0.52*	0.48*
	23-h	0.67*	0.37*	0.42*
1-hydroxypyrene	Pre	0.11	0.14	0.12
	3-h	0.56*	0.45*	0.47*
	6-h	0.81*	0.33*	0.29*
	12-h	0.73*	0.26*	0.23*
	23-h	0.49*	0.23*	0.22*
Hydroxyfluorenes	Pre	0.34	0.35	0.32
	3-h	1.1*	0.74*	0.67*
	6-h	0.61*	0.53*	0.44*
	12-h	0.44*	0.40*	0.39*
	23-h	0.36	0.34	0.33

^a^Hydroxynaphthalenes = Σ 1-hydroxynaphthalene and 2-hydroxynaphthalene; hydroxyphenanthrenes = Σ 1-hydroxyphenanthrene, 2-hydroxyphenanthrene, and 3-hydroxyphenanthrene; hydroxyfluorenes = Σ 2-hydroxyfluorene and 3-hydroxyfluorene

^b^Bolded and italicized values represent peak excretion

*Significantly different from pre-exposure levels (*p* < 0.05)

The pre to 3-h post-firefighting change in OH-PAH metabolite concentrations for the attack and search firefighters was compared by attack tactic (Table 2). Compared to transitional attack, interior attack resulted in higher increases of hydroxyfluorenes, 1-hydroxypyrene, and hydroxyphenanthrenes (p-values comparing the difference were <0.01, 0.09, and 0.052, respectively). In contrast, hydroxynaphthalenes increased slightly more for the transitional attack.

**Table 2:** Median pre- to 3-h post-exposure change in urinary OH-PAH metabolites for 24 firefighters assigned to attack and search (2 fires each, different tactic for each fire) stratified by fire-attack tactic

Attack tactic	*n*	OH-NAP	OH-PHE	1-PYR	OH-FLU
Interior	24	+382%	+657%	+342%	**+160%**
Transitional	24	+411%	+418%	+172%	**+128%**
*p*-value^a^		0.229	0.052	0.091	**0.008**

^a^Bolded values are statistically significant at *p* < 0.05

We found statistically significant (*p* < 0.05) correlations between the non-specific PAH metabolites (measured via ELISA) and each specific OH-PAH metabolite (measured via HPLC-MS/MS) at pre-exposure (except for 2-hydroxynaphthalene) and 3-hr post-firefighting (Table S1, supplemental materials). Overall, the strongest correlations were for the 3-h collection (Spearman r ranging from 0.31 to 0.53). These results suggest that the non-specific PAH metabolite concentrations measured using ELISA approximated the more specific OH-PAH metabolites during the critical pre and 3-hr post-firefighting collections. The pre- to 3-h post-firefighting change in non-specific PAH metabolites was statistically significant for the attack and search firefighters, increasing a median of 1.7-fold (*p* < 0.01), for the outside vent firefighters, increasing a median of 1.3-fold (*p* = 0.02), and for the overhaul firefighters, increasing a median of 1.2-fold (*p* = 0.05). We did not find statistically significant pre- to 3-h post exposure changes for firefighters assigned to command/pump. In the subset of the population where additional post-exposure samples were collected over 23 h (Table 3), the 6-hr collection represented peak excretion for the attack and search and outside vent firefighters and 12-h collection represented peak excretion for the command/pump and overhaul firefighters.

**Table 3:** Summary of ELISA PAH metabolite concentrations (µg/g phenanthrene equivalents) in 36 firefighters (distributed evenly among 3 crews) assigned to attack, search, outside vent, backup/overhaul, RIT/overhaul, and command/pump who were sampled for 23 h after a single interior fire attack scenario

Job assignment	Collection period	Median^a^	IQR
Attack/search (*n* = 12)	Pre	26.6	22–41
	3-h	50.1	38–69
	6-h	***57.5***	52–69
	12-h	42.8	38–62
	23-h	38.1	30–52
Outside vent (*n* = 6)	Pre	23.1	18–34
	3-h	30.0	19–50
	6-h	***35.9***	28–46
	12-h	29.7	19–38
	23-h	33.7	26–40
Overhaul (*n* = 12)	Pre	32.5	25–39
	3-h	41.4	31–48
	6-h	43.0	34–58
	12-h	***44.9***	25–55
	23-h	39.0	25–45
Command/pump (*n* = 6)	Pre	26.9	26–34
	3-h	30.2	19–43
	6-h	32.7	30–47
	12-h	***37.3***	31–44
	23-h	38.7	29–52

^a^Bolded and italicized values represent peak excretion

### Exhaled breath concentrations of VOCs after firefighting

**Fig. 2:**
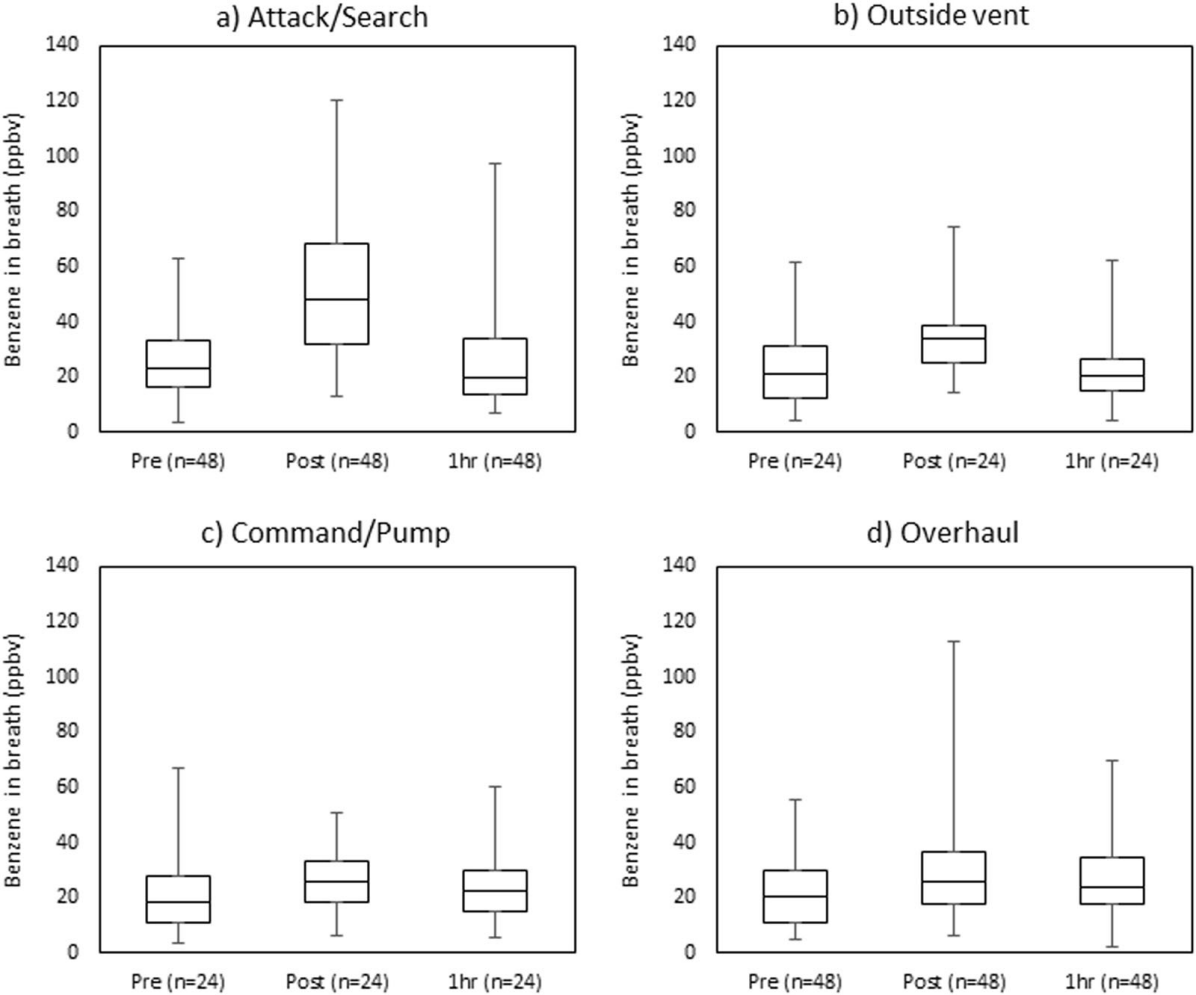
Exhaled breath concentrations of benzene (ppbv) over time for 36 firefighters (distributed evenly among 3 crews, 4 fires per crew) assigned to **a** attack or search, **b** outside vent, **c** command/pump, or **d** backup/overhaul or RIT/overhaul. Note that firefighters were reassigned after first two fires. Immediate post-firefighting concentrations were statistically different from pre-firefighting concentrations (*p* *<* 0.05) for firefighters assigned to attack and search, outside vent, and overhaul

The pre- to immediate post-firefighting change in breath concentrations of benzene, toluene, ethylbenzene, styrene, and xylenes were calculated for each job assignment (Table S2, supplemental materials). Median exhaled breath concentrations of benzene increased after firefighting (Fig. 2) and this increase was statistically significant (*p* < 0.05) for attack and search (2-fold increase, *p* < 0.01), outside vent (1.4-fold increase, *p* = 0.01), and overhaul (1.3-fold increase, *p* = 0.02). Somewhat surprisingly, firefighters in all job assignments had decreased median concentrations of xylenes and ethylbenzene in their breath after firefighting, and these changes were statistically significant (*p* ≤ 0.01) for firefighters assigned to attack and search. No difference in breath concentrations of benzene was found by tactic (interior attack 2-fold increase vs. transitional attack 1.9-fold increase, *p* = 0.63).

During this study, investigators documented when the attack and search firefighters encountered the smoke plume prior to breathing from SCBA (before entering the structure) such as while pulling the attack line from the engine and possibly during exterior water application on the transitional fire attack scenarios. This exposure period, albeit typically very brief (<1 min), could contribute to the biological uptake. This potential inhalation exposure occurred in 20 of the 48 person-events. According to the analysis of these data, the change in exhaled breath concentrations of benzene in firefighters with this exposure (median 1.7-fold increase) and those without this exposure (median 2.3-fold increase) did not differ significantly (*p* = 0.08) nor in the hypothesized direction.

Two firefighters assigned to attack and search had to remove their SCBA before arriving at the breath collection area because they ran out of air. It is possible that these firefighters could have inhaled benzene that was off-gassing from their turnout gear prior to providing their breath sample. However, their post-exposure breath results did not appear to differ from the overall data distribution, suggesting that any contribution from off-gassing gear was insignificant compared to the levels absorbed during firefighting.

## Discussion

This study provides a better understanding of how firefighters’ biological absorption of combustion products varies by fireground job assignment and fire attack tactic. Higher PAH biomarkers and benzene concentrations were found among firefighters assigned to fire attack and search operations than any other job assignment. This is a particularly important finding because the attack and search firefighters protected their airways during the fire response by using SCBA, and most did not remove their SCBA until they reached the breath collection area. Although some of these firefighters could have had inhalation exposure prior to structure entry when SCBA was worn (due to environmental smoke, *n* = 20) or when removing their SCBA post-response (due to off-gassing turnout gear, *n* = 2) [43, 44], we found no difference in exhaled breath concentrations of benzene by these factors. Thus, the findings here support previous work suggesting that dermal absorption plays an important role in the accumulation of toxicants during firefighting [16, 18, 19].

The ability of turnout gear to attenuate PAHs and other combustion byproducts from contacting skin may depend on many factors including physical state (particulate vs. vapors) and concentrations of the airborne chemicals, as well as the properties of the turnout gear (e.g., permeability and tightness of interfaces). Wingfors et al. [15] reported higher turnout gear/station gear workplace protection factors (WPFs) for total PAHs (WPF = 146) than naphthalene (WPF = 49), the most volatile PAH that exists predominantly in gas phase during firefighting. In a previous publication, our group reported median personal air concentrations of benzene for the attack and search firefighters of 40 and 38 ppm, respectively [5]. Median personal air concentrations of total PAHs for the attack and search firefighters were 23.8 and 17.8 µg/m^3^, respectively, and naphthalene accounted for ~50% of the total [5]. Given the high concentrations of benzene and PAHs in the fire atmosphere, even a small percentage of penetration through or around the interfaces of turnout gear could result in toxicologically relevant levels on skin. For the same study, median levels of PAHs on the hands of attack and search firefighters were found to increase from non detectable levels pre-firefighting to 135 and 226 µg/m^2^, respectively after the response [20]. In contrast to the airborne composition, naphthalene made up a small percentage of the total PAHs on hands (median = 8.4%), whereas phenanthrene and pyrene constituted 27 and 23% of the total, respectively [20]. These studies indicate that firefighters’ skin exposure will vary by type of compound and may not correlate directly with the air concentrations.

Studies have shown that a small percentage of benzene vapor (<1%) may be absorbed directly through skin and up to 20–56% of PAHs applied to skin can be absorbed within about 6 h [23, 28, 29]. Factors like skin thickness, skin temperature, sweat, and relative humidity can affect the skin absorption [23, 27, 29]. Residence time on the skin can also affect the magnitude of chemical absorption. In these scenarios, firefighters cleaned their skin and showered soon after fire suppression (within ~45 min). Had contamination been allowed to remain on the skin for longer periods, it is possible that more would have been absorbed.

For the attack and search firefighters, hydroxynaphthalenes had the largest pre to 3-hr post-firefighting unit increase in urine (+27.2 µg/g creatinine), while hydroxyphenanthrenes had the largest relative increase over the same time period (6.6-fold increase). Likewise, benzene was significantly elevated in the breath immediately after the exercise. PAH metabolites measured in urine are primarily the lower molecular weight (MW) compounds (4 rings or less), which are generally less toxic. For example, naphthalene—while classified as an IARC Group 2B carcinogen—is not considered as toxic as benzo[a]pyrene (IARC Group 1) [7, 45]. Benzo[a]pyrene and many of the other higher MW PAHs are primarily excreted in feces. Consequently, 1-hydroxypyrene in urine is often used as a surrogate for these higher MW PAHs. As with the other PAH metabolites, we found marked increases in 1-hydroxypyrene after firefighting, especially in the attack and search firefighters. Interestingly, 1-hydroxypyrene appeared to have a longer excretion profile than the other PAH metabolites. Li et al. [46] determined excretion patterns of PAHs by collecting urine from study subjects after they ingested barbeque chicken and found maximal excretion of 1-hydroxypyrene at 5.5 h vs. 3.1–3.9 h for hydroxynaphthalenes and hydroxyfluorenes (hydroxyphenanthrenes reached maximal excretion at 4.1–5.3 h). This study also found that 24–48 h were required for the OH-PAH metabolites to return to background. This may explain why the participants’ pre-firefighting median concentrations of OH-PAH metabolites were 1.1 to 2-fold higher than the general non-smoking adult population medians [42]. While prolonged excretion (>23 h) was found in the attack and search firefighters, it is important to note that metabolism and excretion of PAHs inhaled or absorbed through skin may vary from ingestion.

For perspective, we compared the firefighters’ 3-h post-firefighting urine OH-PAH concentrations to other occupations. In a review of PAH exposure in various occupations, Huang et al. [47] found that workers who handled coal tar products generally had the highest urinary 1-hydroxypyrene concentrations, with averages ranging from 0.4–334 µg/g creatinine for coke oven operators, 0.3–7.7 µg/g creatinine for gas workers, and 1.2–3.5 µg/g creatinine for road pavers. The 1-hydroxypyrene concentrations 3-h after firefighting (e.g., attack/search median = 0.86 ug/g creatinine, maximum = 2.6 µg/g creatinine) were generally well below the upper mean ranges reported for coke oven operators and gas workers and just below the upper mean ranges for road pavers.

Several studies have investigated firefighters’ exposures to PAHs [15, 16, 30, 31, 48, 49]. Keir et al. [16] studied firefighters’ exposures during emergency fire responses and found average post-event (18-h integrated urine sample) increases of 3.7 fold for 1-hydroxypyrene, 5.3-fold for hydroxyphenanthrenes, 2.9-fold for hydroxynaphthalenes, and 3.9-fold for hydroxyfluorenes. These increases are remarkably similar to the attack and search firefighters’ 3-h after firefighting in this study (2.3, 5.6, 3.9, and 1.4-fold increases, respectively). When urine was collected over 12-h, urinary concentrations of 1-hydroxypyrene for attack and search firefighters peaked at a 5.8-fold increase 6-h after firefighting (median of 0.81 µg/g creatinine). Wingfors et al. [15] measured a 7.6-fold increase in 1-hydroxypyrene in firefighter students who suppressed wood wool and chipboard training fires, and found that several other PAH metabolites also increased significantly. Caux et al. [30] measured the excretion of 1-hydroxypyrene over a 24-h period following emergency fire responses and found maximum excretion 4–8 h later with median concentrations of 0.3 µg/g creatinine (range: 0.06–7.0 µg/g creatinine). These studies appear to corroborate our findings with respect to the magnitude and timing of PAH metabolite excretion in firefighters’ urine.

The PAH metabolite results here suggest that attack and search firefighters who operate in the interior of the structure are the most exposed, followed by firefighters assigned to outside vent and overhaul. Concentrations of PAH metabolites in the command/pump personnel were only measured using ELISA. Those data show an insignificant increase 3-h after firefighting (1.03-fold increase vs. pre, *p* = 0.21), but a significant modest increase 6-h after firefighting (1.3-fold increase vs. pre, *p* = 0.02). The ELISA results appear to have a longer excretion profile (peak excretion ≥6 h after firefighting) than the HPLC-MS-MS results, perhaps because the ELISA method is non-specific and responds to a variety of PAHs and related compounds [34]. Some of these compounds could have longer half-lives than hydoxylated napthalenes, phenanthrenes, fluorenes, and pyrene. Interestingly, the results for exhaled breath concentrations of benzene by job assignment appear to mirror the urinary PAH metabolite findings with the exception that command/pump personnel had higher median breath concentrations of benzene than the overhaul firefighters. Exposures for the command/pump personnel would primarily be through the inhalation route, which could explain this finding.

For many of the VOCs in breath, concentrations decreased post firefighting. One explanation for this finding is that the SCBA provided cleaner air to breathe than the ambient outdoor air. This, coupled with the fact that median air concentrations of toluene (0.064 ppm), ethylbenzene (0.001 ppm), and xylenes (0.00064 ppm) inside the structure during the fires paled in comparison to benzene (14 ppm), may have resulted in lower exposures to these compounds than what was encountered before testing [5]. On the other hand, the command/pump personnel did not wear SCBA and their concentrations of toluene, ethylbenzene and xylenes also decreased post firefighting. Further discussion of the breath sampling results and interpretation are provided in Wallace et al. [50].

Transitional fire attack is used as a method of effectively suppressing fires initially from the exterior which may also minimize some of the risks associated with direct interior attack [51]. However, it has been proposed that transitional attack could also lessen firefighters’ exposures. In previous work, our group found no statistical differences between personal air concentrations of PAHs or benzene by attack tactic [5]. In the same study, there were no statistical differences in dermal exposures to PAHs by attack tactic; however, median values measured on firefighters’ hands and necks were slightly higher for interior attack [20]. Peak neck skin temperatures in the attack and search firefighters were slightly but significantly higher after using the interior attack compared to transitional [35], which may increase transdermal absorption rate of some chemicals [27]. The urine measurements reported in this current paper indicate that transitional attack resulted in 20%, 36%, and 50% lower systemic levels of hydroxyfluorenes, hydroxyphenanthrenes, and 1-hydroxypyrene, respectively, than interior attack for the attack and search firefighters. This reduction is substantial, especially considering that hydroxyphenanthrenes had the largest percent increase in attack and search firefighters and that 1-hydroxypyrene may best represent the more toxic PAHs. The mechanism leading to these findings is unknown, but appears not to be related to the magnitude of airborne contaminants. Applying water to the fire from the outside will likely change the environmental conditions (e.g., decrease temperature, increase moisture) [35], which in turn could change the composition and physical state of the combustion byproducts. For example, as the environment cools, airborne contaminants may partition more heavily into the particle phase, where turnout gear may provide better attenuation [15]. It is important to note that there was no effect of tactic on the exhaled breath concentrations of benzene. However, benzene will likely remain in gas phase even after the environment cools. Overall, our findings suggest that transitional attack could be used as an administrative control to reduce firefighters’ exposures to PAHs when it may be appropriate considering the entirety of the fireground needs. It is important to note that selection of fire attack tactics must consider a broad range of factors in addition to firefighters’ exposures.

In this paper, PAH metabolite results measured using an ELISA method were presented mainly as a supplement to the HPLC-MS-MS results. The advantages of this method are that it is relatively inexpensive and responds well to a variety of different PAH compounds [34]. The disadvantages of this method are that it is non-specific, generally not as sensitive as mass-spectrometry methods, and liable to miss important patterns and associations. ELISA results were significantly correlated with the majority of the pre-firefighting and 3-h post-firefighting HPLC-MS-MS results, but this did not hold true for the later collections. Future studies that use the ELISA method must take these limitations into account.

Other limitations of this study are that the measured PAHs and VOCs only represent a small percentage of the combustion products generated during a fire. Risk assessments based on these results alone may underestimate the true hazard from exposures during structural firefighting. Such risk assessments should consider the potential for additive or synergistic effects from the mixture of compounds encountered during modern structure fires, as well as the potential for exposure beyond the fire scene (e.g., contaminants on turnout gear, in the apparatus, and in the firehouse bay and living quarters). Despite these limitations, this study adds to the understanding of firefighters’ biological uptake of toxicants during structural firefighting and elucidates the effect of job assignment and fire attack tactic on the biological levels. This information may be valuable for the fire service to devise policies and procedures to manage firefighters’ exposures.

## Supplementary information

Supplementary Materials

Supplementary Figure legend

Supplementary FigureS1
