# Supplementary material for: Firefighters’ absorption of PAHs and VOCs during controlled residential fires by job assignment and fire attack tactic
Source: J Expo Sci Environ Epidemiol. 2019 Jun 7;30(2):338–49. doi: 10.1038/s41370-019-0145-2 (PMC7323473; doi:10.1038/s41370-019-0145-2)
Supplement: Supplementary file 1 — Supplementary Materials [file 41370_2019_145_MOESM1_ESM.docx]

**Supplemental Materials**

**Firefighters’ absorption of PAHs and VOCs during controlled residential fires by job assignment and fire attack tactic**

Kenneth W. Fent^1^, Christine Toennis^2^, Deborah Sammons^2^, Shirley Robertson^2^, Stephen Bertke^1^, Antonia Calafat^3^, Joachim D. Pleil^4^, M. Ariel Geer Wallace^4^, Steve Kerber^5^, Denise Smith^6,7^, Gavin P. Horn^7^

1. Division of Surveillance, Hazard Evaluations, and Field Studies, National Institute for Occupational Safety and Health (NIOSH), Centers for Disease Control and Prevention (CDC), Cincinnati, OH, USA
2. Division of Applied Research and Technology, NIOSH, CDC, Cincinnati, OH, USA
3. Division of Laboratory Services, National Center for Environmental Health, CDC, Atlanta, GA, USA
4. National Exposure Research Laboratory, Office of Research and Development, U.S. Environmental Protection Agency, Research Triangle Park, NC, USA
5. Firefighter Safety Research Institute, Underwriters Laboratories, Columbia, MD, USA
6. Skidmore College, Saratoga Springs, NY, USA
7. Illinois Fire Service Institute, University of Illinois at Urbana-Champaign, IL, USA

**Tables**

| **Table S1** Spearman correlation coefficients^a^ between PAH metabolites measured by ELISA and specific OH-PAH metabolites measured by HPLC-MS-MS for the selected job assignments. | | | | | | | | |
| --- | --- | --- | --- | --- | --- | --- | --- | --- |
| Collection period | n | 1-NAP | 2-NAP | 1-PHE | 2,3-PHE | 1-PYR | 2-FLU | 3-FLU |
| Pre-exposure | 96 | **0.39** | 0.18 | **0.36** | **0.36** | **0.27** | **0.41** | **0.41** |
| 3-hr post-exposure | 96 | **0.39** | **0.31** | **0.49** | **0.49** | **0.53** | **0.49** | **0.46** |
| 6-hr post-exposure | 24 | 0.37 | 0.24 | **0.42** | 0.30 | 0.35 | 0.06 | 0.14 |
| 12-hr post-exposure | 24 | 0.28 | 0.22 | 0.17 | 0.10 | 0.15 | 0.21 | 0.23 |
| ^a^ Bolded values are statistically significant at *p* < 0.05. | | | | | | | | |

| **Table S2** Median pre- to post-firefighting percent change^a^ in exhaled breath concentrations of VOCs by job assignment | | | | | |
| --- | --- | --- | --- | --- | --- |
| Job assignment | Benzene | Toluene | Ethyl benzene | Styrene | Xylenes |
| Attack / Search | **105%** | 7.8% | **-25%** | 4.6% | **-32%** |
| Outside vent | **40%** | -2.5% | -19% | 14% | -12% |
| Command / Pump | 33% | -14% | -27% | -18% | -22% |
| Overhaul | **28%** | -6.4% | -10% | 0.5% | -3.5% |
| ^a^ Bolded values are statistically significant at *p* < 0.05. | | | | | |
