# Supplementary material for: Firefighters’ absorption of PAHs and VOCs during controlled residential fires by job assignment and fire attack tactic
Source: J Expo Sci Environ Epidemiol. 2019 Jun 7;30(2):338–49. doi: 10.1038/s41370-019-0145-2 (PMC7323473; doi:10.1038/s41370-019-0145-2)
Supplement: Supplementary file 2 — Supplementary Figure legend [file 41370_2019_145_MOESM2_ESM.docx]

**Supplemental Materials**

**Figure Legends**

**Fig. S1** Study design flow chart showing how participants were distributed and assigned for each of the 4 fire scenarios and the timing, collection, and analysis of biological samples.
