# Supplementary figures and images for: Firefighters’ absorption of PAHs and VOCs during controlled residential fires by job assignment and fire attack tactic
Source: J Expo Sci Environ Epidemiol. 2019 Jun 7;30(2):338–49. doi: 10.1038/s41370-019-0145-2 (PMC7323473; doi:10.1038/s41370-019-0145-2)

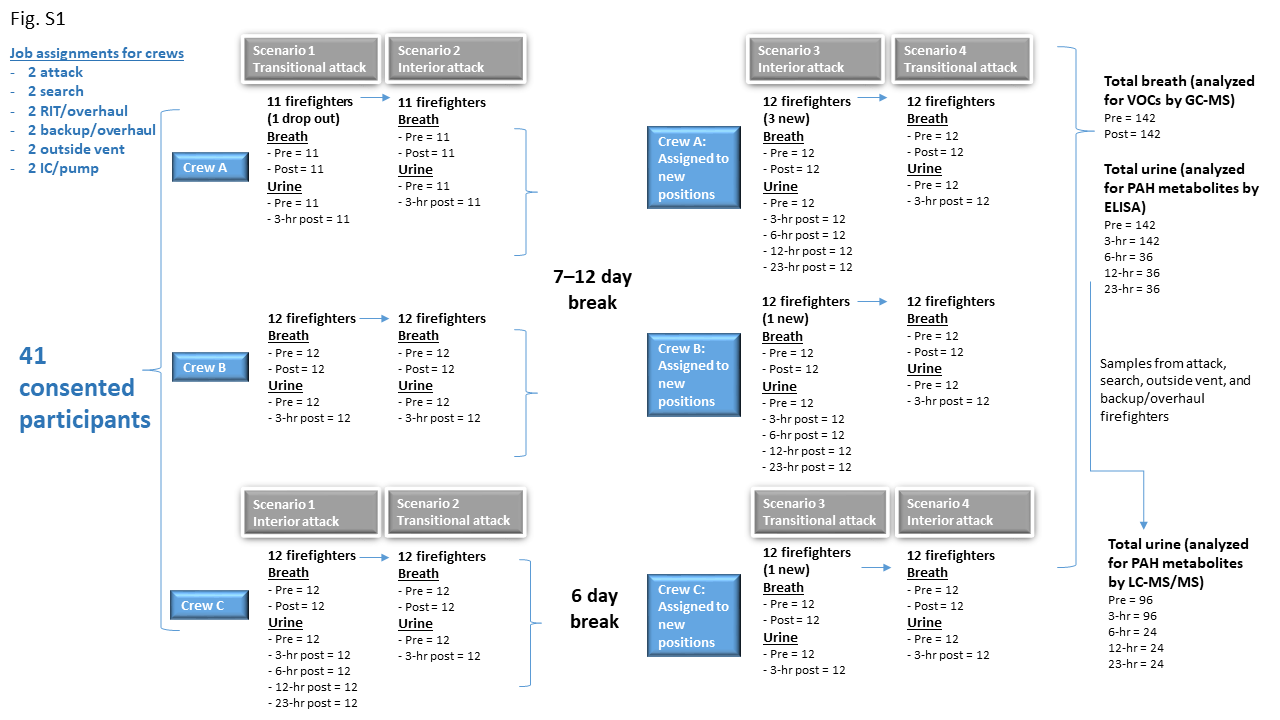

Supplement: Supplementary file 3 — Supplementary FigureS1 [file 41370_2019_145_MOESM3_ESM.tif]
